# Prognostic value of calcium score and coronary flow velocity reserve in asymptomatic diabetic patients

**DOI:** 10.1186/s12947-015-0035-2

**Published:** 2015-09-04

**Authors:** Miodrag Dikic, Milorad Tesic, Zeljko Markovic, Vojislav Giga, Ana Djordjevic-Dikic, Jelena Stepanovic, Branko Beleslin, Ivana Jovanovic, Ana Mladenovic, Jelena Seferovic, Miodrag Ostojic, Aleksandra Arandjelovic

**Affiliations:** Clinic for Cardiology, Clinical Center of Serbia, Visegradska 26, 11000 Belgrade, Serbia; Medical School, University of Belgrade, Belgrade, Serbia; Clinic for Radiology, Clinical Center of Serbia, Belgrade, Serbia; Clinic for Endocrinology, Clinical Center of Serbia, Belgrade, Serbia; Cardiology Department, Clinical Hospital Zvezdara, Belgrade, Serbia

**Keywords:** Coronary artery disease, Calcium score, Coronary flow velocity reserve, Adverse events

## Abstract

**Background:**

The risk stratification of patients with diabetes mellitus (DM) is a major objective for the clinicians, and it can be achieved by coronary flow velocity reserve (CFVR) or with coronary artery calcium score (CS). CS evaluates underlying coronary atherosclerotic plaque burden and CFVR estimates both presence of coronary artery stenosis and microvascular function. Consequently, CFVR may provide unique risk information beyond the extent of coronary atherosclerosis.

**Aim:**

Our aim is to assess joint prognostic value of CFVR and CS in asymptomatic DM patients.

**Materials and methods:**

We prospectively included 200 asymptomatic patients (45,5 % male, mean age 57,35 ± 11,25), out of which, there were 101 asymptomatic patients with DM and 99 asymptomatic patients without DM, but with one or more conventionally risk factors for coronary artery disease. We analyzed clinical, biochemical, metabolic, inflammatory parameters, CS by Agatston method, transthoracic Doppler echocardiography CFVR of left anterior descending artery and echocardiographic parameters.

**Results:**

Total CS and CS LAD were significantly higher, while mean CFVR was lower in diabetics compared to the nondiabetics. During 1 year follow-up, 24 patients experienced cardio-vascular events (one cardiovascular death, two strokes, three myocardial infarctions, nine new onsets of unstable angina and nine myocardial revascularizations): 19 patients with DM and five non DM patients, (*p* = 0,003). Overall event free survival was significantly higher in non DM group, compared to the DM group (94,9 % vs. 81,2 %, *p* = 0,002 respectively), while the patients with CS ≥200 and CFVR <2 had the worst outcome during 1 year follow up in the whole study population as well as in the DM group. At multivariable analysis CFVR on LAD (HR 12.918, 95 % CI 3.865–43.177, *p* < 0.001) and total CS (HR 13.393, 95 % CI 1.675–107.119, *p* = 0.014) were independent prognostic predictors of adverse events in DM group of patients.

**Conclusion:**

Both CS and CFVR provide independent and complementary prognostic information in asymptomatic DM patients. When two parameters are analyzed together, the risk stratification ability improves, even when DM patients are analyzed together with non DM patients. As a result, DM patients with CS ≥200 and CFVR <2 had the worst outcome. Consequently, the use of two tests identified subset of patients who can derive the most benefit from the intensive prevention measures.

## Introduction

The leading cause of mortality in patients with diabetes is coronary artery disease (CAD). Patients with diabetes mellitus (DM) have increased risk of myocardial infarction and cardiovascular death due to accelerated coronary atherosclerosis, thus the risk stratification of DM patients is a major objective for the clinicians [1]. Traditional cardiovascular risk factors such as age, systolic blood pressure, smoking, cholesterol levels and family history can’t completely account for the increased cardiovascular mortality in patients with diabetes [2]. Also novel additional factors like inflammatory factors (fibrinogen, C-reactive protein, Galectin-3) and metabolic factor (lipoproteins) could contribute in explanation of atherosclerosis in diabetes [3–5].

Patients with DM are often asymptomatic until the onset of acute coronary event [6] and they have a poor prognosis [7]. Risk stratification of DM patients with unknown or suspect CAD can be achieved by stress echocardiography [8–13], coronary flow reserve [14–17], or with coronary artery calcium score (CS) [18, 19].

Coronary flow velocity reserve (CFVR) reflects both - the presence of epicardial coronary artery stenosis and function of microcirculation [20–22]. Impaired CFVR in DM patients is occasionally observed in the absence of significant epicardial coronary arteriosclerosis, even in young adults with DM type I of shorter duration without associated CAD [8, 14, 16, 22].

CS has emerged as promising tool for CAD risk assessment [18]. The amount of CS on cardiac computed tomography has been found to correlate with a total coronary atherosclerotic burden and the risk of adverse cardiovascular outcomes [19]. Recent large prospective study demonstrated that the CS is significant independent predictor of cardiovascular events and can enhance the predictive power of traditional cardiovascular risk factors in asymptomatic patients with diabetes [19]. On the other hand, with the CS result should be cautious since, compared to the computed tomography angiography, it can miss non-calcified plaque components [19].

CS evaluates underlying coronary atherosclerotic plaque burden and CFVR estimates both presence of coronary artery stenosis and microvascular function. Consequently, CFVR may provide unique risk information beyond the extent of coronary atherosclerosis. Thus, our aim is to assess joint prognostic value of CFVR and CS in asymptomatic DM patients.

## Methods

### Patients selection

We prospectively included 200 asymptomatic patients (45,5 % male, mean age 57,35 ± 11,25), from October 2012 to the March 2013. Out of 200 patients, there were 101 asymptomatic patients with DM diagnosed by standard criteria and on standard diabetic therapy, including diet, tablets, or insulin and 99 asymptomatic patients without DM, but with one or more conventionally risk factors for coronary artery disease.

Patients with a prior history of CAD (including hospital admission for chest pain, acute coronary syndromes, stable angina or prior coronary revascularization) or any other heart disease were excluded from the study in both groups. Also criteria for exclusion from the study were as follows: poor echocardiographic window for CFVR assessment, chronic obstructive pulmonary artery disease, atrioventricular block, inability to provide informed consent.

We pooled biochemical, metabolic parameters (cholesterol, low-density lipoprotein, high-density lipoprotein, triglycerides, glucose level, hemoglobin A1c (HbA1c), microalbuminuria, inflammatory markers of atherosclerosis (high sensitive C reactive protein and fibrinogen). CFVR of left anterior descending artery (LAD) and multi slice computed tomography (MSCT) of coronary arteries with CS calculation according to Agatston method [23] were done in all patients.

### Echocardiography and coronary flow velocity reserve

Echocardiographic studies were performed with an available digital ultrasound system (Acuson Sequoia C256; Siemens Medical Solutions USA, Inc, Mountain View, CA).

To visualize distal part of LAD artery we used modified, foreshortened, three chamber view, obtained by sliding the 3V2C multifrequency (4-MHz) transducer on the upper part and medially from an apical three chamber view [20, 24]. For color-Doppler flow mapping, the velocity range was set to the range of 16–24 cm/sec. Alignment of the ultrasound beam direction with the distal LAD flow was kept as parallel as possible. All the subjects had Doppler recordings of the LAD at rest and during intravenously slow application of adenosine (0.14 mg/kg/min), during 2 min. By placing the sample volume on the color signal, spectral Doppler of the LAD showed the characteristic biphasic flow pattern (larger diastolic and smaller systolic components). Coronary diastolic peak velocities were measured at baseline and during the intravenous infusion of adenosine by averaging three consecutive Doppler signals for each measurement. CFVR was defined as ratio of hyperemic and baseline diastolic peak velocities [20, 22, 24]. Digital images were obtained and stored on magneto-optical discs and analyzed offline by two well trained physicians.

### Calcium score assessment

All study participants agreed and underwent serial coronary artery CS assessment using MSCT (SOMATOM Sensation 64, Siemens Medical Solutions, Forchheim, Germany) scanner. Heart images view obtained with 100 ms scan time, using 3 mm slices, starting at the level of carina and proceeding to the level of diaphragm (40–45 slices). Tomography imaging was electrocardiographically triggered at 40 % or 65 % of the R-R interval, depending on the subject’s heart rate. CS was defined as a plaque of at least 2 pixels (area 0.67 mm2) with density ≥130 Hounsfield units. Quantitative CS was calculated using Agatston method [23], which involves the multiplication of the area of calcified focus by a cofactor based on the peak density of the lesion. The total CS calculation was done by adding the individual scores of the all lesions found along the entire coronary artery tree [23]. Then, according to the total CS results, patients were grouped into the five CS categories: 0–10, 11–100, 101–400, 401–1000, and >1000 Agatston units, as previously used [19, 25].

Standard invasive coronary angiography was recommended to selected patients according to the current Guidelines by the referred physician. All diagnostic procedures were done at Clinic for cardiology and Clinic for radiology of Clinical Center of Serbia.

Informed consents were obtained for all the participants; the study was in adherence to the tents of Declaration of Helsinki and the local ethical committee approved the study protocol.

### Follow-up data

During a 1 year follow up of the patients; outcomes were determined from patient interviews at the outpatient clinic, hospital chart reviews, and telephone interviews with patients, their close relatives, or referring physician. Cardiovascular death, stroke, nonfatal myocardial infarction, new onset of unstable angina and clinically driven percutaneous coronary intervention (PCI), or coronary bypass grafting (CABG) were registered as clinical events. Diagnosis of death from cardiovascular causes were obtained from the results of postmortem examination and detailed hospital documentation. Myocardial infarction was defined by typical symptoms, electrocardiographic and cardiac enzyme changes, while stroke was defined as rapid onset of focal or global neurological deficit lasting ≥24 h or leading to death, with clinical findings supplemented by neurological imaging. Unstable angina was diagnosed on the basis of clinical features of an acute coronary syndrome without diagnostic enzyme changes or need for hospital admission or both. Follow-up data were analyzed for the prediction of composite endpoint of major adverse cardiovascular and cerebrovascular events (MACCE).

### Statistical analyses

Descriptive statistics were used to summarize baseline demographic, biochemical and clinical characteristics, and risk factors in two groups - DM and non DM group. Categorical variables were compared using chi-square test. Continuous variables were compared by using Simple *T*-test, and/or ANOVA-test, or Kruskal Wallis test (for variables without normal distribution). Statistical correlation among CS and CFVR was examined with the Spearman’s correlation coefficient. Survival rates were estimated with Kaplan-Meier curves and compared by the log-rank test. The association of selected variables with outcome was assessed with the Cox proportional hazard model using univariate and stepwise multivariate procedures. A significance of 0.05 was required for a variable to be included into the multivariate model, whereas 0.1 was the cut off value for exclusion. Hazard ratios with the corresponding 95 % confidence intervals were estimated. The sensitivity and specificity of CFVR and CS for outcome prediction were evaluated with receiver operating characteristic curves. Analyses were performed using SPSS for Windows version 22 (SPSS, Inc, Chicago, IL).

## Results

Demographic, biochemical and clinical characteristics of the study population were presented in Table 1. Mean age of the whole study population was 57,7 ± 11,84 years (60,34 ± 10,21 years in diabetic and 55,04 ± 12,81 years in non diabetic group, *p* < 0.001). Subjects were obese with mean body mass index 27,39 ± 4,73 kg/m^2^ (28,74 ± 4,68 kg/m^2^ in DM group and 26,01 ± 4,39 kg/m^2^ in non DM group, *p* < 0.001). Average systolic blood pressure for the whole population was 128,03 ± 11,44 mmHg (130,10 ± 11,27 mmHg in DM group and 125,91 ± 11,28 mmHg in non DM group, *p* = 0,009).

**Table 1:** Demographic, biochemical and clinical characteristics of the study population

Variables		Non DM	DM	Total	*p* value: non DM vs. DM
		(*n* = 99)	(*n* = 101)	(*n* = 200)	
Male, no. (%)		42 (42,4 %)	49 (48,5 %)	91 (45,5 %)	*p* = 0,387
Age		55,04 ± 12,81	60,34 ± 10,21	57,7 ± 11,84	*p* < 0,001
Obesity	BMI ≤25	52 (52,5 %)	20 (19,8 %)	72 (36,0 %)	*p* < 0,001
	BMI >25	47 (47,5 %)	81 (80,2 %)	128 (64,0 %)	
BMI (kg/m2)		26,01 ± 4,39	28,74 ± 4,68	27,39 ± 4,73	*p* < 0,001
Smoking History, no. (%)	Current	28 (28,2 %)	19 (18,8 %)	47 (23,5 %)	*p* = 0,261
	Former	16 (16,2 %)	21 (20,8 %)	37 (18,5 %)	
	Non smoker	55 (55,56 %)	61 (60,40 %)	116 (58,00 %)	
Family history, no. (%)		65 (65,70 %)	70 (69,30 %)	135 (67,50 %)	*p* = 0,582
HTA, no. (%)		54 (55,70 %)	86 (85,10 %)	140 (70,70 %)	*p* < 0,001
Systolic blood pressure (mmHg)		125,91 ± 11,28	130,10 ± 11,27	128,03 ± 11,44	*p* = 0,009
Hypercholesterolemia, no. (%)		52 (53,1 %)	72 (72 %)	124 (62,6 %)	*p* = 0,006
Cholesterol (mmol/l)		5,54 ± 0,93	5,36 ± 0,91	5,45 ± 0,92	*p* = 0,160
LDL (mmol/l)		3,40 ± 0,87	3,26 ± 0,81	3,33 ± 0,84	*p* = 0,253
HDL (mmol/l)		1,38 ± 0,33	1,19 ± 0,26	1,28 ± 0,31	*p* < 0,001
Triglycerides (mmol/l)		1,52 ± 0,80	2,10 ± 1,12	1,82 ± 1,02	*p* < 0,001
Glucose (mmol/l)		5,37 ± 0,74	8,31 ± 2,76	6,86 ± 2,51	*p* < 0,001
HbA1C (%)		5,51 ± 0,81	7,13 ± 1,32	6,33 ± 1,36	*p* < 0,001
CRP (mg/l)		2,13 ± 1,71	2,89 ± 3,47	2,51 ± 2,77	*p* = 0,141
Fibrinogen (g/l)		3,63 ± 1,04	3,58 ± 1,05	3,61 ± 1,05	*p* = 0,734
Microalbuminuria, no. (%)		0 (0,00 %)	26 (25,7 %)	26 (13,0 %)	*p* < 0,001

Plus–minus values are means ± SD, *BMI* body mass index, *CRP* C-reactive protein, *DM* diabetes mellitus, *LDL* low-density lipoprotein, *HDL* high-density lipoprotein, *HbA1C* hemoglobin A1c, *HTA* hypertension

There were no significant differences between DM and non DM group in gender, prevalence of smoking and family history of cardiovascular disease, level of cholesterol, low-density lipoproteins, high sensitive C-reactive protein and fibrinogen level (Table 1). Diabetic patients had significantly more often hypertension, microalbuminuria, higher systolic blood pressure, lower level of HDL, higher level of triglycerides, glucose, HbA1C (Table 1).

CFVR and CS parameters were presented in Table 2. There were no differences between groups in baseline and hyperemic velocities of LAD, but mean value of CFVR was significantly lower in DM group (*p* = 0,011). The percent of patients with pathological values of CFVR <2 was significantly higher in diabetics compared to the nondiabetics (18,8 % vs. 3,0 %, *p* < 0,001, respectively). Total CS and CS LAD were significantly higher in diabetics compared to the non DM group. In our study there was significantly higher rate of CS 0–100 in non diabetics (74,8 % vs. 44,6 %), while higher rate of CS ≥401 was found in diabetics (20,8 % vs. 5,0 %). In subgroup of patients with cut off of CS ≥200 Agatston units there was significantly higher rate of diabetics compared to non diabetics (44,6 % vs.15,2 %, *p* < 0,001 respectively).

**Table 2:** Coronary flow velocity reserve and calcium score parameters

Variables		Non DM	DM	Total	*p* value: non DM vs. DM
		(*n* = 99)	(*n* = 101)	(*n* = 200)	
Baseline diastolic flow velocity (m/sec), LAD		0,27 ± 0,05	0,27 ± 0,07	0,27 ± 0,06	*p* = 0,663
Hyperemic diastolic flow velocity (m/sec), LAD		0,72 ± 0,16	0,67 ± 0,19	0,70 ± 0,18	*p* = 0,061
Rest heart rate		71,27 ± 10,86	70,70 ± 9,11	70,98 ± 10,0	*p* = 0,687
Hyperemic heart rate		76,23 ± 12,36	75,23 ± 10,82	76,06 ± 11,58	*p* = 0,835
CFVR		2,65 ± 0,41	2,48 ± 0,44	2,56 ± 0,44	*p* = 0,011
CFVR <2		3 (3,0 %)	19 (18,8 %)	22 (11,0 %)	*p* < 0,001
CFVR ≥2		96 (97,0 %)	82 (81,2 %)	178 (89,0 %)	
CS - total		93,10 ± 196,59	322,76 ± 534,84	209,09 ± 419,56	*p* < 0,001
CS - LAD		46,16 ± 93,24	142,32 ± 232,88	96,24 ± 184,40	*p* < 0,001
CS, no. (%)	0–10	56 (56,6 %)	31 (30,7 %)	87 (43,5 %)	*p* < 0,001
	11–100	18 (18,2 %)	14 (13,9 %)	32 (16,0 %)	
	101–400	20 (20,2 %)	35 (34,6 %)	55 (27,5 %)	
	401–1000	3 (3,0 %)	14 (13,9 %)	17 (8,5 %)	
	≥1001	2 (2,0 %)	7 (6,9 %)	9 (4,5 %)	
CS 0–199		84 (84,8 %)	56 (55,4 %)	140 (70,0 %)	*p* < 0,001
CS ≥200		15 (15,2 %)	45 (44,6 %)	60 (30,0 %)	

Plus–minus values are means ± SD, *DM* diabetes mellitus, *LAD* left anterior descending artery, *CS* calcium score, *CFVR* coronary flow velocity reserve, *CVD* cardiovascular disease

There were significant correlations between CFVR LAD and total CS (*r* = −0,266, *p* = 0.008) as well as with CS of LAD artery (*r* = −0,249, *p* = 0.013) in DM patients. CFVR and CS did not correlate significantly in non DM patients. There was also no relation between HbA1c and CFVR of LAD in our DM population.

During 1 year follow-up, 24 patients (12 %) experienced cardio-vascular events: 19 patients with DM (17,8 %) and five patients (5,1 %) in non DM group,(*p* = 0,003), Table 3.

**Table 3:** Major adverse cardiac and cerebrovascular events, calcium score and coronary flow velocity reserve in patients with and without diabetes mellitus

		Non DM		DM		Total	%	*p* value: non DM vs DM
		(*n* = 99)		(*n* = 101)		(*n* = 200)		
		Number	%	Number	%	Number		
MACCE	No	94	94,9 %	82	81,2 %	176	88 %	*p* = 0,003
	Yes	5	5,1 %	19	18,8 %	24	12 %	
CS/MACCE	0–199	1	20 %	1	5,3 %	2	8,3 %	*p* = 0,289
	≥200	4	80 %	18	94,7 %	22	91,7 %	
CFVR/MACCE	CFVR <2	1	20 %	15	78,9 %	16	66,7 %	*p* = 0,013
	CFVR ≥2	4	80 %	4	21.1 %	8	33,3 %	
Events	Death	0	0,0 %	1	5,3 %	1	4,2 %	*p* = 0,321
	MI	1	20 %	2	10,5 %	3	12,5 %	*p* = 0,573
	PCI	1	20 %	6	31,6 %	7	29,2 %	*p* = 0,050
	CABG	0	0,0 %	2	10,5 %	2	8,3 %	*p* = 0,159
	Stroke	1	20 %	1	5,3 %	2	8,3 %	*p* = 0,989
	Unstable Angina	2	40 %	7	36,8 %	9	37,5 %	*p* = 0,987
Mean follow-up –months		15,44 ± 3,38		14,14 ± 5,95				
		(14,46–17,84)		(12,96–15,32)				
Overall survival		94,9 % ± 2,2 %		81,2 % ± 3,8 %				

Plus–minus values are means ± SD, *CS* calcium score, *CFVR* coronary flow velocity reserve, *DM* diabetes mellitus, *MACCE* major adverse cardiac and cerebrovascular event, *MI* myocardial infarction, *PCI* percutaneous coronary intervention, *CABG* coronary arteries bypass grafting

When we analyzed type of events, there were significantly higher number of PCIs in diabetic group compared to non diabetic group (31,6 % vs. 20 %, *p* = 0,050 respectively). There were no significant differences in death, MI, CABG and onset of angina between two groups. Using a receiver operating characteristic analysis, CFVR = 2 and CS = 200 were the best predictors of future events (area under the curve = 0,801, sensitivity = 66,7 %, specificity = 83,0 % and area under the curve = 0,928, sensitivity = 91,7 %, specificity = 79,2 %, respectively) and were taken as cut off values for the further statistics.

**Fig. 1:**
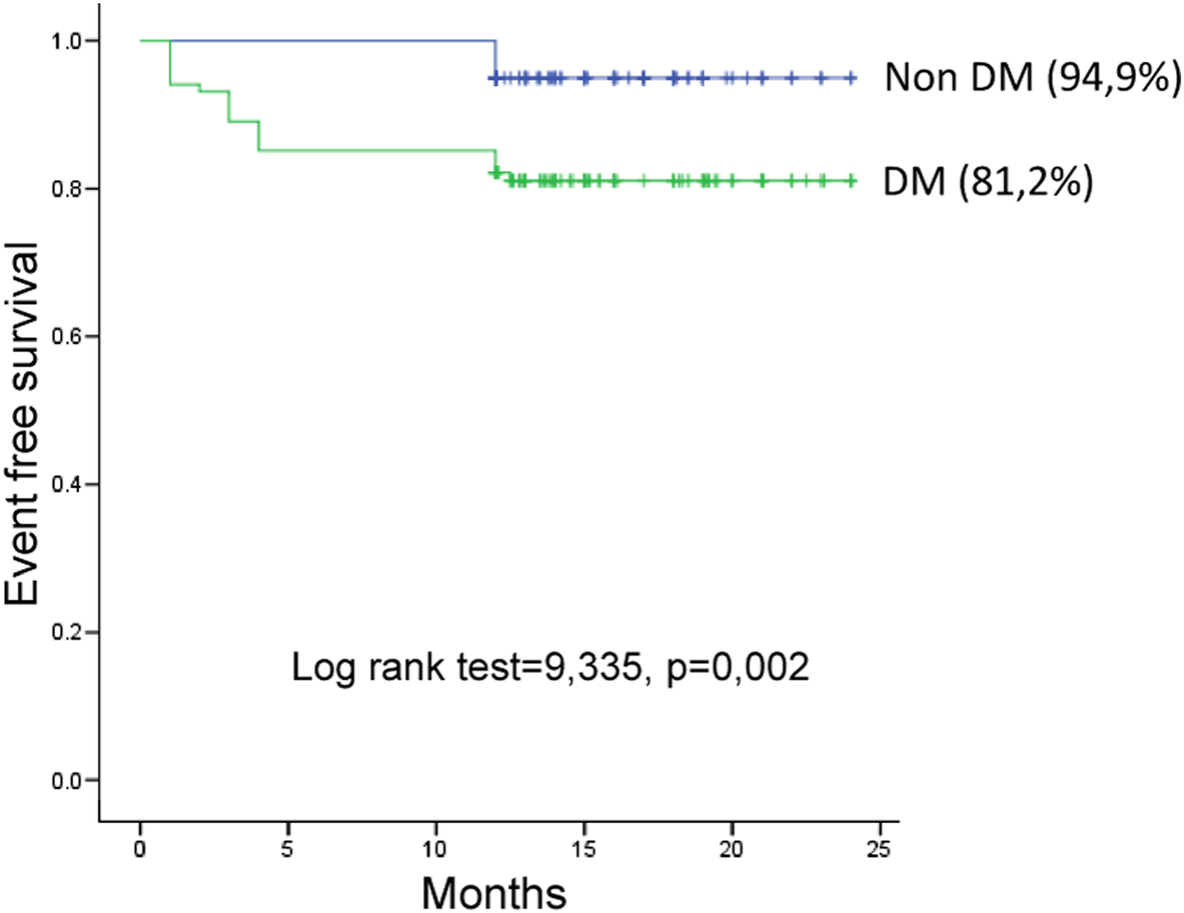
Kaplan-Meier event-free survival curve in patients with and without diabetes mellitus. DM: diabetes mellitus

**Fig. 2:**
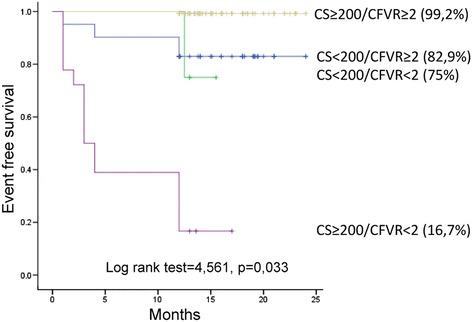
Kaplan-Meier event-free survival curve in diabetic and non diabetic patients stratified according to the presence of CS <200 or CS ≥200 and CFVR <2 or CFVR ≥2. The worst survival is observed in patients with CS ≥200 and CFVR <2. CS: calcium score, CFVR: coronary flow velocity reserve

**Fig. 3:**
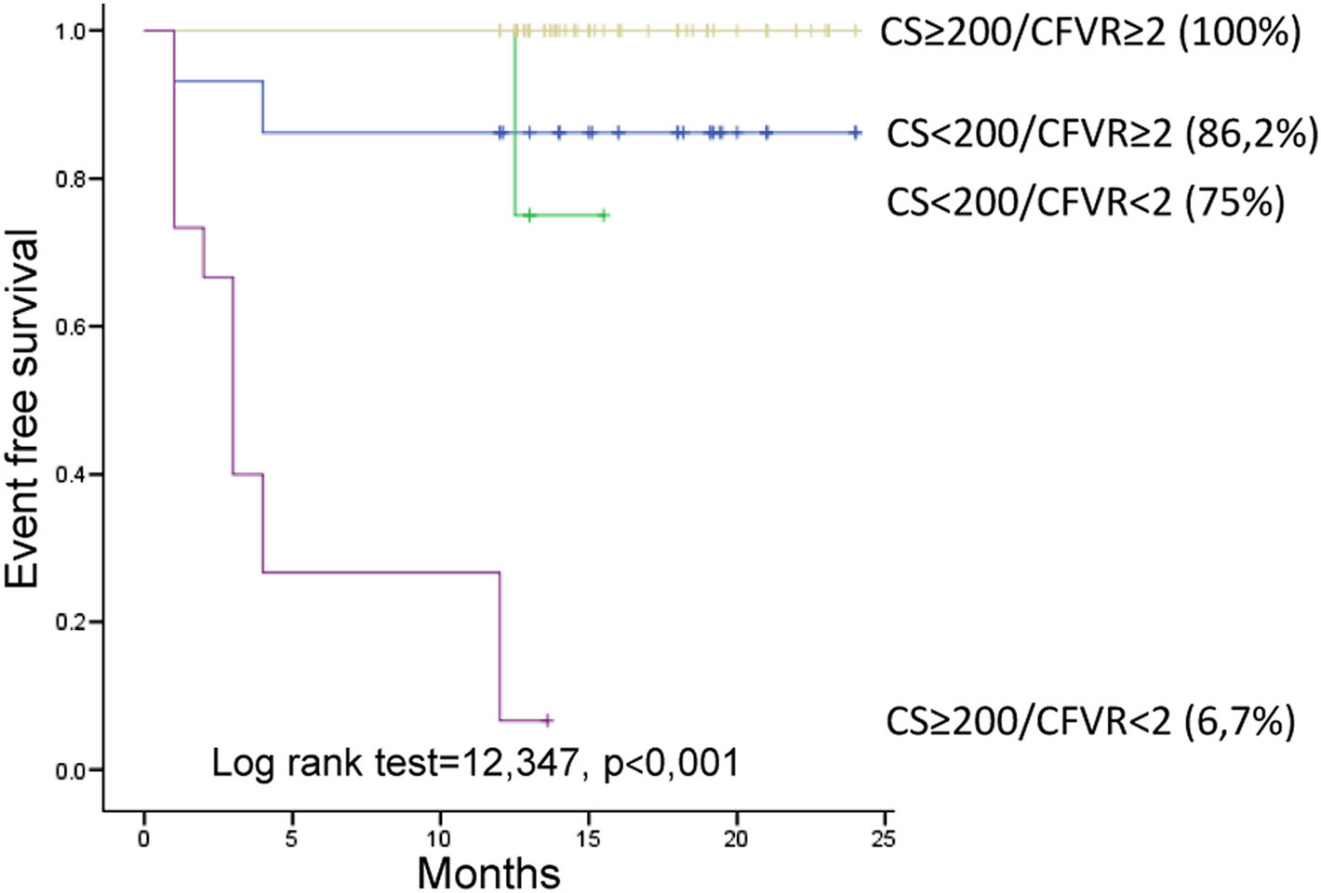
Kaplan-Meier event-free survival curve in diabetic patients stratified according to the presence of CS <200 or CS ≥200 and CFVR <2 or CFVR ≥2. The worst survival is observed in patients with CS ≥200 and CFVR <2. CS: calcium score, CFVR: coronary flow velocity reserve

Overall event free survival was significantly higher in non DM group, compared to the DM group (94,9 % vs. 81,2 %, *p* = 0,002 respectively),as presented in Fig. 1, while the patients with CS ≥200 and CFVR <2 had the worst outcome during 1 year follow up in the whole study population (Fig. 2), as well as in the DM group (Fig. 3).

In the whole study population patients with CS ≥200 and CFVR <2 had a 24,3 fold increase in cardiovascular risk compared to the patients with CS <200 and CFVR ≥2 (95 % CI: 5,13–106,97; *p* < 0.001), while in the DM group, patients with CS ≥200 and CFVR <2 had 43,7 fold increase in cardiovascular risk compared to the patients with CS <200 and CFVR ≥2 (95 % CI: 7,09–269,77; *p* < 0,001).

Univariate predictors of MACCE are reported in Table 4. At multivariable analysis CFVR on LAD (HR 12.918, 95 % CI 3.865–43.177, *p* < 0.001) and total CS (HR 13.393, 95 % CI 1.675–107.119, *p* = 0.014) were independent prognostic predictors of MACCE in DM group of patients as presented in Table 4.

**Table 4:** Univariate and multivariate prognostic predictors of adverse events in diabetic patient

	Univariate analysis				Multivariate analysis			
	HR	95.0 % CI for HR		*p*	HR	95.0 % CI for HR		*p*
		Lower	Upper			Lower	Upper	
Gender (male)	4,365	1,448	13,162	0,009	1,431	0,455	4,498	0,539
Age	1,863	0,671	5,173	0,233				
Obesity	0,970	0,322	2,925	0,958				
HTA	1,602	0,370	6,937	0,528				
Family history of CAD	0,948	0,360	2,494	0,913				
Smoking habit	0,891	0,351	2,263	0,808				
Hypercholesterolemia	2,533	1,028	6,239	0,043	1,149	0,441	2,990	0,777
Microalbuminuria	1,713	0,674	4,351	0,258				
CRP	1,503	0,604	3,736	0,381				
Fibrinogen	1,100	0,447	2,706	0,863				
CS (≥200<)	26,707	3,559	200,381	0,001	13,393	1,675	107,119	0,014
CFVR (≥2<)	24,240	7,944	73,970	<0,001	12,918	3,865	43,177	<0,001

*CRP* C-reactive protein, *HTA* hypertension, *CS* calcium score, *CFVR* coronary flow velocity reserve, *CAD* coronary artery disease, *HR* hazard ratio, *CI* confidence interval

## Discussion

This study, represents the results of the annual follow up regarding the prognostic value of CS and CFVR in asymptomatic diabetic patients, where abnormal CFVR <2, detected by Doppler echocardiography identified together with CS ≥200 subset of patients at higher risk for adverse events. When the two parameters are analyzed together, the risk stratification ability improves, even when DM patients are analyzed together with non DM patients. Thus, our data confirms and expands previous studies, suggesting not only that CFVR and CS have prognostic value when separately analyzed but also that the combination of the two parameters has additive value and that they are complementary in their power of prediction. In our study, we found that older male as well as obese patients, mostly diabetics, have higher level of CS. At the same time, asymptomatic diabetic patients had significantly higher both total CS and CS of LAD artery compared to the control group which is consistent with published studies [25–27].

The presence of diabetes carries a higher risk, and studies have shown that CS 0 can be helpful to stratificate these subjects into the low-risk category, with a lower rate of adverse events and excellent survival. In asymptomatic individuals with DM, prevalence of CS 0 in patients without diabetes was twice as high as compared to the subjects with diabetes with the same CS, as similar to our study, but there was no difference in survival during 5 years follow up (98.8 % vs. 99.4 %, *p* = 0,5) [25]. Meta-analysis of 18 studies showed that CS 0 had a negative predictive value of 93 % for the presence of obstructive CAD, suggesting that CS 0 can safely exclude presence of obstructive CAD [28]. Other studies also showed that patients with CS = 0, have very low risk of cardiac events during the follow-up as well as in our study [29, 30]. In the study with asymptomatic DM patients during 8-years follow-up, it was presented that the CS over 400 had a significantly higher prevalence of cardiovascular events compared to the group with lower CS (5.6 % vs. 0.7 %, *p* < 0.01) [31]. It was also shown, that CS increased in proportion to an adverse events from 0 to 18 % when the score rised from 100 to 1000 Agatston units [31]. We presented that diabetics with CS ≥200 had more often MACCE, which is consistent with the results of published studies [31, 32]. Prediction of adverse cardiovascular events in asymptomatic DM patients compared according to the value of CS and followed during 4 years, was recently presented in prospective cohort study [19]. Authors analyzed classical risk factors, as well as metabolic and inflammatory factors (lipoprotein, apolipoprotein, homocysteine and C-reactive protein) [19]. They showed that the CS is independent predictor of the cardiovascular events [19]. We also analyzed inflammation (CRP and fibrinogen), and metabolic (glycated hemoglobin, hypercholesterolemia and microalbuminuria) parameters but we found that the CFVR and CS were the only independent predictors of adverse events.

As previously showed, we found that CFVR LAD was significantly lower in DM group compared to the non DM group [14, 15, 22]. Nemes et al. determined the prognostic impact of diabetes and CFVR in patients with suspected coronary artery disease. During 41 ± 12 months they recorded 22 cardiovascular deaths (13 sudden cardiac death, seven acute heart failure, two had cardiovascular thrombosis) [17]. Multivariable regression analysis showed that only CFVR and DM were independent predictors of cardiovascular survival [17]. According to the ROC analysis, the best predictive value of CFVR was 1.73 [17]. In contrast to this study, our study examined asymptomatic patients with and without diabetes and had only one death of DM patient during the shorter follow up, while the best predictor value of CFVR for the adverse events was two. Cortigiani et al. furthermore point out importance of evaluation of microcirculation in DM patients without severe CAD (≤50 % diameter coronary stenosis), where CFVR ≤2 represented a strong and independent predictor of the combined event of death and nonfatal MI predicting a nearly seven times higher yearly hard-event rate compared with preserved CFVR [14]. In the same study, nonobstructive CAD failed to provide independent prognostic contribution, although it was associated with significantly lower mean CFVR [14].

Reduced CFVR in DM patients without coronary artery disease is due to functional and structural changes of coronary microcirculation [33–35]. It is also known that coronary microcirculatory dysfunction can reduce CFVR, even without inducing regional wall motion abnormalities or before the occurrence of coronary artery stenosis, but can still be linked with adverse events in patients with DM or LV hypertrophy [8, 9, 14, 24]. Microvascular dysfunction affects LV globally [14], thus the CFVR LAD is excellent option for evaluation of microcirculation due to high feasibility (94–98 %) in various studies [8, 14, 20, 24]. Therefore we found that in asymptomatic DM patients the assessment of CFVR as a marker of function of both micro- and macro-circulation together with evaluation of coronary morphology by CS is a reasonable diagnostic method for the risk stratification of adverse coronary events.

### Study limitations

There were some limitations in our study. First we enrolled asymptomatic diabetic patients, and asymptomatic controls according to their anamnestic data that they don’t have any anginal symptoms and fatigue. Our study is relatively small, because there were just 200 subjects enrolled out of which 101 DM patients. Also we did not have age matched control group, but we believe that eventhough the age was significantly different between groups that difference cannot affect significantly values of CFVR and CS [36]. Although measurement of CFVR in all three coronary arteries is preferable, CFVR was measured only in the LAD due to technical challenge in the present time to visualize all three coronary arteries with high feasibility [24]. We didn’t focus on diastolic dysfunction, which is reported in diabetes, and leads to the impaired CFVR as well as hypertension. However in both groups we had patients with hypertension, which contributed to the results of CFVR in both groups.

## Conclusion

Both CS and CFVR obtained by MSCT and by transthoracic Doppler echocardiography assessments, respectively, provide independent and complementary prognostic information in asymptomatic DM patients. When the two parameters are analyzed together, the risk stratification ability improves, even when DM patients are analyzed together with non DM patients. As a result, DM patients with CS ≥200 and CFVR <2 had the worst outcome. Consequently, the use of two tests identified subset of patients who can derive the most benefit from the intensive prevention measures - more aggressive control of the risk factors and more frequent follow-up by noninvasive testing.

## Abbreviations

DM: Diabetes mellitus
non DM: Control group
AS: Agatston score
MACCE: Major adverse cardiovascular and cerebrovascular events
HTA: Hypertension
CRP: C-reactive protein
CAD: Coronary artery disease
CFVR: Coronary flow velocity reserve
CI: Confidence interval
CS: Calcium score
LAD: Left anterior descending coronary artery
MSCT: Multidetector computed tomography
MI: Myocardial infarction
PCI: Percutaneous coronary intervention
CABG: Coronary bypass grafting
